# Relevance of ERK1/2 Post-retrieval Participation on Memory Processes: Insights in Their Particular Role on Reconsolidation and Persistence of Memories

**DOI:** 10.3389/fnmol.2019.00095

**Published:** 2019-04-17

**Authors:** Maria C. Krawczyk, Julieta Millan, Mariano G. Blake, Mariana Feld, Mariano M. Boccia

**Affiliations:** ^1^Laboratorio de Neurofarmacología de los Procesos de Memoria, Cátedra de Farmacología, Facultad de Farmacia y Bioquímica, Universidad de Buenos Aires (UBA), Buenos Aires, Argentina; ^2^Instituto de Fisiología y Biofísica (IFIBIO UBA-CONICET), Facultad de Medicina, Universidad de Buenos Aires (UBA), Buenos Aires, Argentina; ^3^CONICET-Universidad de Buenos Aires, Instituto de Fisiología, Biología Molecular y Neurociencias (IFIBYNE), CABA, Argentina

**Keywords:** ERK/MAPK, inhibitory avoidance, memory reconsolidation, memory persistence, PD098059, mice

## Abstract

Back in 1968, Misanin and his group posited that reactivation of consolidated memories could support changes in that trace, similar to what might happen during the consolidation process. Not until 2000, when Nader et al. (2000) studied the behavioral effect of a protein synthesis inhibitor on retrieved memories, could this previous statement be taken under consideration once again; suggesting that consolidated memories can become labile after reactivation. The process of strengthening after memory labilization was named memory reconsolidation. In recent years, many studies pointed towards a critical participation of the extracellular signal-regulated kinase (ERK)/mitogen activated protein kinases (MAPKs) pathway in different memory processes (e.g., consolidation, extinction, reconsolidation, among others). In this review article, we will focus on how this system might be modulating the processes triggered after retrieval of well-consolidated memories in mice.

## Introduction

New memories are initially vulnerable to disruption but are progressively strengthened over time. The phenomenon is known as memory consolidation (McGaugh, 2000; Dudai and Eisenberg, 2004). Throughout the past century and the beginning of this one, several studies have shown that retrieved memories, conscious- or unconsciously, can be reformulated and updated (Eysenck, 1976; Spear et al., 1990; Przybyslawski and Sara, 1997; Nader et al., 2000; Carbó Tano et al., 2009; Dudai, 2012; Forcato et al., 2014; Fernández et al., 2017). This new process was named after memory reconsolidation. In order to reconsolidate, a memory must first undergo a time-dependent destabilization process after retrieval-induced reactivation (Misanin et al., 1968; Sara, 2000; Nader et al., 2000; Dudai and Eisenberg, 2004). During this period, memory becomes susceptible to modulation by different interventions such as the administration of: agonists or antagonists, inhibitors of molecular pathways or a new training situation (Przybyslawski and Sara, 1997; Roullet and Sara, 1998; Przybyslawski et al., 1999; Nader et al., 2000; Milekic and Alberini, 2002; Pedreira and Maldonado, 2003; Pedreira et al., 2004; Suzuki et al., 2004; Romano et al., 2006; Boccia et al., 2007, 2010; Tronson and Taylor, 2007; Baratti et al., 2009; Blake et al., 2013; Exton-McGuinness et al., 2015; Krawczyk et al., 2015; Krawczyk et al., 2016).

The ability to generate changes implies, first of all, that memory should be in a labile state (active) and, secondly, that the re-stabilization process of this trace occurs in order to allow changes to persist over time (reconsolidation). Now, if every time an experience is recorded, labilization and subsequent reconsolidation might be triggered, then memory might suffer irreversible damage such as experience removal. Thus, why certain conditions or requirements are necessary in order for the reconsolidation process to take place (e.g., presentation of a specific reminder of the previously stored experience)? In this framework, Lewis (1979) showed that not every time a memory is reactivated, and consequently goes into an active state, will it be modified. Then, the mechanism responsible for the re-stabilization of the trace after memory reactivation would hold on specific conditions: the strength of the unconditioned stimulus (US) used during training (Boccia et al., 2004; Suzuki et al., 2004), the age of the memory (Milekic and Alberini, 2002; Boccia et al., 2006), the structure of the reminder: duration of the conditioned stimulus (CS; Pedreira and Maldonado, 2003), mismatch between what is expected and what actually happens (Pedreira et al., 2004) and prediction error (Exton-McGuinness et al., 2015; revised in Krawczyk et al., 2017).

Existence of the reconsolidation process has been proved not only in rodents (Judge and Quartermain, 1982; Przybyslawski et al., 1999; Nader et al., 2000; Boccia et al., 2003, 2004) but in other vertebrate and invertebrate species (Anokhin et al., 2002; Pedreira et al., 2002; Pedreira and Maldonado, 2003; Forcato et al., 2007; Hupbach et al., 2009); suggesting a conserved—evolutionary phenomenon.

There seems to be a close link between memory processes and mental disorders (reviewed in Fernández et al., 2016). Since the impairment of memory reconsolidation precludes the re-storage process, this phase has been proposed as a promising tool for “editing memories”; thus, undermining any potential recovery or generalization of the psychopathology. During the past few years, several translational approaches of memory reconsolidation have shown to be encouraging (Przybyslawski and Sara, 1997; Brunet et al., 2008, 2014; Soeter and Kindt, 2015), hence highlighting the importance of further studying the subcellular mechanisms underlying this process.

Most research studies about memory reconsolidation processes have used tasks in which a CS is paired with an US during training session. Therefore, subjects learn that the CS predicts the outcome of the US. Commonly, no repetition of CS-US pairing is used on the retrieval session (CS is presented in the absence of US), which might allow the triggering of at least two distinct memory processes: reconsolidation or extinction. In this scenario, extinction could be considered as a new memory (CS with no US) which would need to undergo a consolidation process in order to stabilize and endure as a long-term memory (LTM; Myers and Davis, 2002). By contrast, reconsolidation would be considered as a reformulation or update of the previous consolidated memory, undergoing a new de-stabilization/stabilization period.

Much is known about LTM and the consolidation process. We can define LTM as those that are stored for long periods of time, being able to persist for days, weeks, months, years or even for the animal’s whole life (McGaugh, 2000). One question we might ask is what makes memory endure for a lifetime? Which mechanisms and brain structures are involved in this process? Several investigators addressed these questions over the past years, studying the persistence of consolidated memories using different tasks and looking into different brain regions (Frankland et al., 2001, 2004; Tischmeyer et al., 2003; Burwell et al., 2004; Cui et al., 2004; Maviel et al., 2004; Bekinschtein et al., 2007, 2008b; Federman et al., 2013; Katche et al., 2016; Villar et al., 2017).

We now know that memory consolidation, reconsolidation and persistence are protein synthesis-dependent processes, which ultimately contributes to synaptic plasticity. Although they might share some molecular pathways (e.g., brain-derived neurotrophic factor–BDNF, IGF2, activity-regulated cytoskeleton-associated protein—Arc/Arg3.1, among others) and involve almost the same brain areas, they have different critical time-windows (Bekinschtein et al., 2008a; Bevilaqua et al., 2008; Medina et al., 2008; Dudai, 2012) and they are certainly not identical (Taubenfeld et al., 2001; Lee et al., 2004; Alberini et al., 2006; Tronson and Taylor, 2007).

In this review, we will focus on how extracellular signal-regulated kinase (ERK)/mitogen activated protein kinases (MAPKs) pathway might be considered as a critical step in the reconsolidation/persistence processes taking place after memory reactivation.

## Memory Reconsolidation and ERK/MAPKs Pathway

The MAPKs play a critical role in the transduction of extracellular signals into intracellular responses (Meloche and Roux, 2012). Over the last 20 years, one of the most studied MAPKs pathways in learning and memory has been the ERK 1/2. Human ERK1 (p44) and ERK2 (p42) are coded by different genes, located in different chromosomes although they share more than 80% identity (Li et al., 2011). These proteins are co-expressed in almost every type of cell and body tissue (Boulton and Cobb, 1991; Boulton et al., 1991; Frémin et al., 2015), recognizing the same target Ser/Thr-Pro sequence in their substrates (Gonzalez et al., 1991).

The interest in the study of ERK 1/2 in plasticity and memory processes comes from the mechanisms that regulate their activation and their substrates. They have been extensively studied in memory processes using different animal models, behavioral tasks and in several brain areas (Bailey et al., 1997; English and Sweatt, 1997; Martin et al., 1997; Kelly et al., 2003; Duvarci et al., 2005; Feld et al., 2005; Miller and Marshall, 2005; Cestari et al., 2006; Boggio et al., 2007; Martijena and Molina, 2012; Zhai et al., 2013; Krawczyk et al., 2015). In this sense, studies carried out in several organisms suggest that the activation of these protein kinases is a necessary and essential biochemical event for the formation of LTM (Martin et al., 1997; Atkins et al., 1998; Crow et al., 1998; Blum et al., 1999). ERK 1/2 activation, *via* phosphorylation, in the hippocampus has been shown to depend on a variety of extracellular signals such as: trophic factors (Gottschalk et al., 1998); glutamate (through NMDAR), β-adrenergic and muscarinic receptor activation (mediated by PKC, PKA or AMPc-dependent mechanisms; de Rooij et al., 1998; Roberson et al., 1999).

Neurotrophic factors are known to play different roles in the regulation of neuronal structure, function, and survival during development and adulthood (Bibel and Barde, 2000; Kaplan and Miller, 2000). Among these, BDNF plays a critical role in long-term synaptic plasticity in the adult brain (Schuman, 1999; Schinder and Poo, 2000). This neurotrophic factor is believed to be the best known transcriptional target of the cAMP-response element binding protein (CREB), emerging as an important synaptic modulator of synaptogenesis (Jiang et al., 2016). Ying et al. (2002) explored for the first time BDNF mechanisms in long-term synaptic plasticity *in vivo*, showing that BDNF triggers long-term potentiation (BDNF-LTP), which requires MEK-ERK activation for its induction (but not for its maintenance). They also described that BDNF-LTP is associated with ERK-dependent activation of CREB and upregulation of the Arc-associated protein immediate early gene (IEG; Arc/Arg3.1). In this scenario, both ERK and CREB emerged as critical points of convergence in the signaling pathways regulating gene transcription in late LTP and LTM (Atkins et al., 1998; Impey et al., 1998; Davis et al., 2000). Furthermore, studies carried on using early life stress paradigms (e.g., maternal separation, restraint, cold shock) report decreases in BDNF levels and concomitant decreases in pERK1 and pERK2 in the hippocampus; being the MAPKs pathway considered as the linking mechanism between classic response to stress signal (glucocorticoids) and BDNF systems (Lemche, 2018).

Among neuronal proteins coded by immediate-early genes (IEGs), *Arc* is unique in that it localizes near recently activated synapses having a crucial role in synaptic plasticity. In this sense, several studies reported that *Arc* is induced by stimuli that also happen to evoke long-lasting synaptic enhancement (LTP) and suppression (LTD); two major forms of synaptic plasticity (Minatohara et al., 2016). Chen et al. (2017) studied the intracellular signaling of *Arc*, providing evidence that NMDAR-mediated ERK and CREB activation plays a critical role in glutamate-induced activation of *Arc* in cortical neurons. Specifically, they found a significant increase in ERK and CREB phosphorylation after glutamate treatment, which was partially prevented by the administration of an NMDAR inhibitor. Another studied IEG protein is Zif268/Egr1 (Zif268). Studies carried on in cell cultures revealed that ERK activation, by phosphorylation, strongly potentiates its ability to activate transcription of certain IEGs through a ternary complex assembled on the serum response element (SRE; Wasylyk et al., 1998). Davis et al. (2000) demonstrated that *in vivo* inhibition of ERK phosphorylation and nuclear translocation in the dentate gyrus in rats prevented LTP-induced transcriptional activation of Zif268.

In the last years, different authors highlighted the role of ERK1/2 in memory reconsolidation processes studying different behavioral tasks (e.g., novel object recognition: Kelly et al., 2003, fear conditioning: Duvarci et al., 2005; Cestari et al., 2006; Martijena and Molina, 2012; Besnard et al., 2013, 2014; Merlo et al., 2014, 2018; and cocaine-addiction models: Miller and Marshall, 2005; Valjent et al., 2006) and different brain areas (e.g., hippocampus: Kelly et al., 2003; Besnard et al., 2013, amygdala: Duvarci et al., 2005; Martijena and Molina, 2012; Merlo et al., 2014, 2018).

### Differential Role of ERK1 and ERK2 in Memory Reconsolidation

The question of whether ERK1 and ERK2 have independent roles from each other or act redundantly has been the subject of intense research and controversy over the years (Boulton et al., 1991). The lack of specific inhibitors for each isoform made the analysis of their function even more difficult. In 2002, Mazzucchelli et al. (2002) proposed a model in which ERK1 would constrain ERK2 function. By studying an ERK knockout mice model, they found that ERK2 (but not ERK1) knockout mice were embryonically lethal; promoting the idea that ERK1 plays an accessory function related to ERK2. When performing behavioral tasks, both ERK1 knockout mice and control mice exhibit successfully learned to avoid punishment by not entering into the dark compartment (“passive avoidance” task) or by running into the opposite compartment when a shock was supposed to be delivered (active avoidance task; Mazzucchelli et al., 2002). Following these experiments, in 2008, Samuels et al. (2008) performed a series of behavioral studies in a conditional ERK2 knock-out mice model. In this case, LTM deficits were found while performing cued and contextual fear conditioning tasks when ERK2 expression was impeded in the CNS (Samuels et al., 2008). Later experiments, with the same conditional ERK2 knockout mice, showed that pharmacological inhibition of ERK1 did not further impair LTM indicating that ERK2 but not ERK1 plays a critical role in its regulation (Satoh et al., 2011). The Yerkes-Dodson law suggests that elevated arousal levels can improve performance up to a certain point (Yerkes and Dodson, 1908). In this regard, in 2012, Maldonado et al. (2011) suggested that selective ERK2 activation in basolateral amygdala (BLA) following stress exposure is a determinant for the stress-induced enhancement effect on fear memory.

So far, the experiments above described studying the distinct role of ERK1 and ERK2 on memory processes. In 2003, Kelly et al. (2003) demonstrated for the first time the participation of hippocampal ERK1/2 in memory reconsolidation processes in rats in a novel object recognition task. In their experiments, animals were injected with vehicle or a MEK inhibitor 40 min before memory reactivation (brief exposure to the objects presented in the training session). Twenty-four hours later, when rats were challenged with a familiar object (same object that both training and retrieval sessions) and a novel object, MEK inhibitor-treated group did not show a preference for the novel object; suggesting a MAP kinase-dependent mechanism of reconsolidation. This effect was dependant on reactivation of the memory trace. When analyzing western blots from the dorsal dentate gyrus and CA1 hippocampal regions, a significant increase in both pERK1 and pERK2 after reexposure to the objects was observed only in CA1.

In 2005, Duvarci et al. (2005) studied the role of ERK1/2 in the lateral amygdala (LA) during reconsolidation of auditory fear conditioning in rats. By administering a MEK inhibitor intra-LA immediately after memory retrieval, they impaired post-reactivation LTM stabilization. No effects were found when memory was not reactivated, suggesting of the need for ERK1/2 pathway during reconsolidation of auditory fear memories in the amygdala. This data was also confirmed in mice in 2006 when Cestari et al. (2006) studied the effects of post-retrieval systemic administration of a MEK inhibitor in ERK1-mutant and control mice in a fear conditioning paradigm. In this case, they observed a decrease in animals’ behavioral response in both mutant- and control-injected groups, suggesting a differential role of ERK2 in memory reconsolidation processes. The decrease in animals’ performance was not observed in the absence of the MEK inhibitor’s infusion. This was the first time that a pharmacological approach was combined with genetic tools to assess the function of ERK/MAPKs in memory reconsolidation processes.

Then, in 2015, our group showed distinct roles of hippocampal ERK1 and ERK2 isoforms in memory reconsolidation using an IA task in mice (Krawczyk et al., 2015). We studied the activation levels of ERK1 and ERK2 after memory reactivation in nuclear and cytosolic hippocampal protein extracts. Next, we studied the effects of an intra-hippocampal administration of a MEK inhibitor immediately after memory reactivation, showing a dose-dependent decrease in performance in a subsequent retention test. This was not observed if the memory was not reactivated or if MEK inhibitor was administered 3 h post-retrieval. Based in our results we proposed that there is a hippocampal cytosolic ERK2 phosphorylation fine tuning specifically during memory reconsolidation, as the activation and posterior inhibition of ERK2 at 15 and 45 min (respectively) after memory reactivation seems to be critical steps in memory re-stabilization.

### Role of ERK 1/2 in Memory Strength After Memory Reactivation

Besnard et al. (2014) studied the relationship between memory and ERK 1/2 phosphorylation patterns after memory formation and reactivation in a contextual fear conditioning paradigm. By using immunocytochemical detection of ERK1/2 pathway they first studied the temporal dynamic of ERK1/2 phosphorylation in different subregions of the hippocampus (dentate gyrus, CA1 and CA3) and amygdala (LA, BLA and CeA) immediately after memory formation or reactivation. They found an increase in the number of dental gyrus and CA3 pERK1/2 immunoreactive cells compared to not trained control groups at different times following both training and retrieval sessions. In the LA and BLA, the number of pERK1/2-positive cells was significantly increased immediately after training (0 and 15 min) and in a delayed manner after retrieval (30 min). Then, they examined the relationship between the strength of a previously established CFC memory and neuronal activity throughout the hippocampus and amygdala by studying the pattern of ERK1/2 phosphorylation immediately after memory retrieval. Animals were trained with one or three footshocks or in the absence of the aversive stimulus (control group). They found an increase in the number of hippocampal (dentate gyrus, CA1 and CA3) but not amygdalar pERK1/2 immunoreactive cells in all three training conditions compared to control groups. Surprisingly, in the dentate gyrus, the highest number of pERK1/2 immunoreactive cells was found in the no-footshock training-condition group suggesting that the presentation of a neutral context triggers the activation of a dentate granule cell population. Besnard and colleagues concluded that it may represent a “background” activity in response to the first re-exposure to a previously explored, but non-reinforced, environment.

In 2016, we provided new evidence about the hippocampal role of cytosolic ERK 1/2 in memory strength in reconsolidation processes (Krawczyk et al., 2016). We studied IA behavior in a one-trial learning, step-through type situation (Boccia et al., 2004). During training (TR), mice received one of two different well-characterized footshock intensities: either low footshock (LFS: 0.8 mA, 50 Hz, 1 s) or high footshock (HFS: 1.2 mA, 50 Hz, 1 s). A third group was also included undergoing no footshock (USh group) as they stepped into the dark compartment (Boccia et al., 2010). These three groups showed significantly different behavioral responses 48 h later in a retrieval session: while HFS animals did not go into the dark chamber, USh animals crossed into it within 10 or less s; and LFS animals expressed an intermediate latency to step through. While the reactivation of IA memory on animals trained with the HFS induced an increase in hippocampal ERK2 cytosolic activation levels at 15 min but a decrease at 45 min after memory reactivation; animals trained with the LFS displayed an increase in their activation levels at both time-points (15 and 45 min) after retrieval. Animals trained in the absence of the US (USh), memory reactivation induced an increment in cytosolic ERK2 activation levels at 15 min, but no changes at 45 min after the retrieval session. Interestingly, in all three training conditions, no changes in hippocampal ERK1 (nuclear or cytosolic) or nuclear ERK2 activation levels were found.

The results above depicted might be in agreement with “background” idea originally set by Besnard (see above; Besnard et al., 2014). In our results, we observed an increase in ERK2 cytosolic activation levels 15 min after memory reactivation in the USh group. The study was performed homogenizing the entire hippocampus, not being able to discriminate which specific hippocampus subareas might be involved in ERK1/2 differential activation levels. Similar results were observed when studying Nf-κB activation levels during memory consolidation and reconsolidation (Freudenthal et al., 2005; Boccia et al., 2007; Salles et al., 2017). First, we described hippocampal Nf-κB activation levels at different time-points after training in both LFS and USH mice in an IA task. The temporal course studied revealed an initial inhibition at 15 min after training with a subsequent activation at 45 min in both SH and USH groups (Freudenthal et al., 2005). We then performed the same analysis after memory reactivation, where both USH and HFS groups showed higher activation levels at 15 min compared to naïve group (Boccia et al., 2007). Taken together, these results may suggest that USH animals formed a memory of their experience during the training session, which is susceptible of being reactivated/labilized by re-exposure to the training context (Krawczyk et al., 2015).

**Figure 1:**
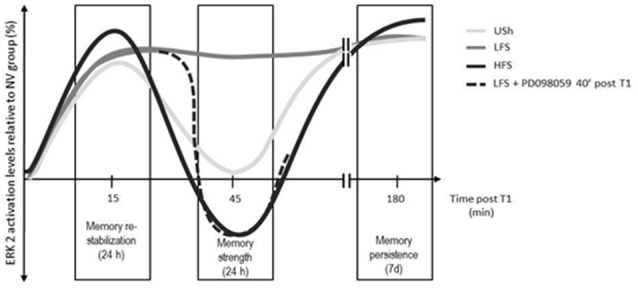
Role of hippocampal cytosolic extracellular signal-regulated kinase 2 (ERK2) in memory reconsolidation (stabilization, strengthening and persistence). Schematic representation of cytosolic ERK2 activation levels at different time points after memory reactivation on mice trained in the IA task. Solid lines represent cytosolic ERK2 activation levels at 15 min, 45 min and 3 h post-retrieval session in animals trained with a low (LFS), high (HFS) footshock intensity or without shock (unshocked, USh). Dashed line represents LFS-trained animals that received a dHIP infusion of PD098059 40 min after the reactivation session ended (T1). ERK2 activation levels between 15 and 45 or between 45 and 180 min have not been determined, but are represented as continuous lines in order to simplify interpretations. T1: reactivation session.

We also evaluated whether the differences in cytosolic ERK2 activation levels at 45 min after retrieval (increase in LFS-trained animals and decrease in HFS-trained animals, both compared to naïve groups) were specific to training conditions. It is noteworthy that, the intra-hippocampal administration of a MEK inhibitor 40 min after the retrieval session in LFS-trained animals, enhanced memory performance in a subsequent retention test 24 h later. The behavioral response was similar to those observed in HFS-trained animals, suggesting that ERK2 cytosolic activation levels at 45 min might be a critical point determining the strength of the retrieved memory (Krawczyk et al., 2015; schematic representation in Figure 1). This was the first report showing a bidirectional regulation of ERK2 during memory reconsolidation of an IA task in mice hippocampus that seems to be driving memory strength.

### Role of ERK1/2 in Different Cellular Compartments

Although different authors highlighted the role of ERK1/2 in memory reconsolidation processes, the majority of the studies were performed without intra-cellular compartment differentiation.

Besnard et al. (2013) examined the precise role of hippocampal ERK1/2 activity and Zif268 gene expression dosage in CFC memory retrieval and reconsolidation by using a Zif268 homozygous and heterozygous mutant mice and an ERK1/2 inhibitor. In their results, Zif268 heterozygous mice displayed a selective impairment of CFC memory reconsolidation only if CFC memory was relatively recently formed and directly reactivated. Furthermore, the administration of an ERK1/2 inhibitor prior to recall decreases recall performance in Zif268 heterozygous mice but a normal performance on a second retention test 1 day later; protecting mice against the deleterious effect of Zif268 gene knockdown on memory reconsolidation. They concluded that besides the known transcriptional role of ERK1/2 activation, it may also have a role in different cellular compartments that leads to rapid post-translational modifications, affecting neuronal excitability and synaptic transmission, independently of transcriptional programs (Besnard et al., 2013).

Taking this final remark, we want to highlight the results obtained from our group, where we studied the role of hippocampal ERK1 and ERK2 in memory reconsolidation processes in both cytosolic and nuclear protein extracts. We observed differences in ERK2 activation levels only in the cytosolic hippocampal protein extracts, meaning that it might be just having a specific role in the cytosolic compartment (although we cannot rule out that our findings might precede nuclear activation). In the molusk *Aplysia californica*, apCAM (a cell adhesion molecule) internalization in sensory neurons has been shown to be related to synaptic growth induced by 5-HT during long-term facilitation. When researchers overexpressed transmembrane constructs with a single point mutation in the two MAPK phosphorylation consensus sites and/or injected a specific MAPK inhibitor into sensory neurons, the internalization process was blocked (Bailey et al., 1997); suggesting that MAPK phosphorylation at the membrane is an important event for apCAM internalization. Thus, it may represent an early regulatory step in the growth of new synaptic connections that accompanies long-term facilitation (Bailey et al., 1997). In this sense, Feld et al. (2005) evidenced the need for cytosolic ERK activation in the crab *Neohelice granulata* (previously called *Chasmagnathus granulatus*), an invertebrate memory model, during consolidation. Together, this body of evidence points to an extra-nuclear key role of ERK2 in memory processes as there has already been demonstrated in other fields of research.

## Reconsolidation-Induced Memory Persistence

Up to date, there is no clear definition or distinction between memory consolidation and persistence. In fact, the term “consolidation” is currently used to describe two distinct types of processes that might be co-existing: one process is fast and dependent on early molecular and cellular events (fulfilled within minutes to hours after training, termed synaptic or cellular consolidation), while the other is slower and involves the interaction among different brain areas such as medial temporal lobes and neocortical structures (takes several days, weeks or even months after training to be completed, termed systems consolidation; Squire et al., 2015). The precise time window for each one is still a matter of discussion.

Little is known about the molecular and cellular events that mediate long-lasting memories or their persistence. It has been found that delayed administration of protein synthesis inhibitors post-training did not alter memory consolidation process. Different waves of protein synthesis were described that might be responsible, at least in part, for memory’s persistence (Bekinschtein et al., 2008b). Assuming that memory’s molecular substrates do not endure over a lifetime (as might be the case for memory); the requirement of new proteins’ synthesis might represent a limitation for memory to persist over time. In this sense, epigenetic mechanisms are thought to be involved in determining memory strength and persistence (Frankland et al., 2006; Miller and Sweatt, 2007; Federman et al., 2009, 2013; Miller et al., 2010; Gräff et al., 2014; Halder et al., 2016; Ding et al., 2017; Zalcman et al., 2019). It was also reported by Rossato et al. (2009), that dopaminergic cells’ activation in the ventral tegmental area (VTA) immediately and 12 h after training might be critically involved in memory persistence; leading to downstream hippocampal BDNF production 12 h after training, a determinant molecular step for long lasting-LTM (Bekinschtein et al., 2008b). This BDNF-induced persistence would also be ERK-dependent, since the administration of an ERK inhibitor 15 min before BDNF infusions (12 h after TR) prevented its effects on long-lasting memories (Bekinschtein et al., 2008b). Hippocampal ERK activity was also found to follow circadian oscillation and pharmacological and physiological interference with these oscillations after memory consolidation was accomplished, impaired its persistence (Eckel-Mahan et al., 2008).

Regarding persistence of reactivated memories, there is scarce evidence about the molecular mechanisms that might underlie this process. To our knowledge, only a few studies address this question (Nakayama et al., 2013; Krawczyk et al., 2016). Nakayama and colleagues first studied the effects of post-retrieval BLA administration of a protein synthesis inhibitor on memory persistence. They showed that the administration of the inhibitor 9.5 h after the reactivation session impaired memory retention performance at 7 days but not 2 days after retrieval of CFC memories (Nakayama et al., 2013). This result was not observed if the injection was 5 or 24 h after retrieval or if the memory was not reactivated, indicating that late-phase BLA infusión of the protein inhibitor after fear memory retrieval attenuates memory persistence in a time- and retrieval-dependent manner.

Then, they studied the BLA Arc expression, known to be required both for consolidation and reconsolidation processes (Ploski et al., 2008; Maddox and Schafe, 2011), at different time-points after CFC memory retrieval (Nakayama et al., 2016). There was a significant increase in Arc expression 2 and 12 h after memory retrieval and these were not observed if the memory was not previously reactivated. The BLA infusion of an Arc antisense 7 h after the re-exposure to the conditioned context altered mice performance at 7 days but not 2 days after memory retrieval. Taking all the results into account, they concluded that Arc late expression (12 h post-retrieval) was essential for the persistence of reactivated fear memories in mice; being a refinement the neuronal circuit through pruning in the BLA a possible mechanism by which late Arc expression contributes to memory persistence.

In the previous section, we discussed hippocampal ERK1/2 participation in memory reconsolidation processes. We concluded that activation and inhibition of ERK2 phosphorylation at different time-points (15 and 45 min) after memory reactivation are critical steps in memory re-stabilization and strengthening (Figure 1). With these in mind, we studied the hippocampal ERK1/2 pattern of activation 3 h after memory reactivation in an IA task in mice. We found that cytosolic but not nuclear activation levels were significantly increased in both isoforms compared to naïve groups, regardless of the training condition (USh, LFS or HFS, Krawczyk et al., 2016). No differences in ERK1/2 activation levels were found if the memory the was not previously reactivated, implying that the increase in ERK1/2 activation levels 3 h post-retrieval was specific to memory reactivation. Next, we examined the effects of 3 h post-retrieval intra-hippocampal administration of a MEK inhibitor in retention performances 1 day or 7 days after memory reactivation. There was no effect on memory retention when animals were tested 1 day after retrieval, suggesting that ERK1/2 participation on memory reconsolidation was enclosed within a time-window shorter than 3 h (Krawczyk et al., 2015). However, when animals were tested 7 days post-retrieval a significant impairment on retention performance was observed in both training conditions (LFS and HFS) only when memory was reactivated, suggesting that this would be a more general mechanism for IA memory persistence in mice. The results above described were the first report showing ERK-induced memory persistence after memory reactivation in mice, independent from the memory consolidation process. Nonetheless, its functional significance and the complete signaling cascade remain an open question and deserve further research.

## Concluding Remarks

Memory reconsolidation is proposed as the mechanism by which memories can be either updated or changed, opening new venues for potential therapeutic treatments and translational ideas on mental disorders (Alberini, 2005; Corlett et al., 2009; Kindt et al., 2009; Schiller et al., 2010; Nader et al., 2013). In this sense, many studies posit the re-consolidation process not only as a mechanism necessary for the maintenance of some psychopathologies, but they also propose it as a novel therapeutic target (Corlett et al., 2009; Taylor et al., 2009; Pitman, 2011; Debiec, 2012; Sevenster et al., 2013; Ecker et al., 2015; Lane et al., 2015; revised in Fernández et al., 2017). Moreover, in the last years, several reports suggested reconsolidation might be considered as a potential therapy mechanism for treating maladaptive addiction-related memories (Xue et al., 2012; Das et al., 2015; Hon et al., 2016; Goltseker et al., 2017). In this sense, ERK/MAPKs pathway has been shown to participate in distinct drug-addiction disorders, allowing the hypothesis that it would contribute to generating long-term stable alterations in synaptic transmission underlying learning and memory (Berhow et al., 1996; Valjent et al., 2000, 2005; Jenab et al., 2005; Ferguson et al., 2006; Edwards et al., 2009; Sun et al., 2015; García-Pardo et al., 2016; Torres et al., 2018). These alterations, according to a recent hypothesis, would ultimately lead to hypersensitivity to drug-associated cues, impulsive decision making and abnormal habit-like learned behaviors that are insensitive to adverse consequences (revised in Lu et al., 2006).

In the present work, we revised the last studies in the literature that examined the importance of different molecular pathways in post-retrieval memory processes. In particular, we discussed the critical role of ERK/MAPKs pathway in the reconsolidation of aversive and appetitive memories. We also opened a new range of questions referring to post-retrieval processes that might contribute to the persistence of reactivated memories. It is important to emphasize that, although so far literature is scarce, ERK1/2 pathway and Arc expression, seem to be critical steps in the persistence of reactivated fear memories. It still remains elusive which are the ERK partners that make possible such a wide spectrum of cellular functions in neuronal populations supporting memory stabilization. The understanding of these processes would be extremely beneficial for potential application to psychiatric disorders.

## Author Contributions

MK, MF, and MMB contributed to the design and implementation of the research, to the analysis of the results and to the writing of the manuscript. JM and MGB contributed to some experiments described in the manuscript.

## Conflict of Interest Statement

The authors declare that the research was conducted in the absence of any commercial or financial relationships that could be construed as a potential conflict of interest.
